# Skin Conductance Responses to a Discrete Threat in Virtual Reality: Associations with Psychopathy and Anxiety

**DOI:** 10.1007/s10862-021-09943-7

**Published:** 2021-12-26

**Authors:** Luna C. M. Centifanti, Steven M. Gillespie, Nicholas D. Thomson

**Affiliations:** 1 Primary Care and Mental Health, University of Liverpool, Liverpool, L69 7ZA UK; 2 Departments of Surgery and Psychology, Virginia Commonwealth University, Richmond, USA

**Keywords:** Psychopathy, Skin conductance, Virtual reality, Anxiety, Fear

## Abstract

People with high levels of psychopathic traits are often described as fearless and lacking in emotional depth, particularly when evaluating threats in their environments. Skin conductance responsivity (SCR) to negative emotional stimuli represents a robust autonomic correlate of conduct problem behavior in children (Fanti et al., in *Neuroscience and Biobehavioral Reviews*, 100, 98–107, 2019). However, studies that have examined threat-related processing in youth with conduct problems have tended to use a variety of negative stimuli that might induce various and unspecific negative emotions. Few studies have taken in to account the moderating effects of anxiety on the relationship of distinct psychopathic traits (e.g., narcissism, callousness, impulsivity) with SCR to a fear inducing stimulus. In this study, we examined SCR to a virtual reality rollercoaster drop – that is, a discrete fear inducing event – in a sample of 75 youths (61 males; *M* = 14 years, *SD* = 1.4) enrolled in a non-mainstream school. The rollercoaster drop was used to more clearly examine an event-related response to a discrete threat, rather than examining SCR throughout the rollercoaster ride. We used the teacher-reported Antisocial Process Screening Device (Frick & Hare, in *Antisocial process screening device: APSD*. Toronto: Multi-Health Systems, 2001) to examine the relations of distinct psychopathic traits with SCR and self-reported anxiety. Lower anxiety was associated with higher callousness, but only in youths with low SCR to discrete threat. These findings suggest that fear and anxiety show complex and interactive relations with distinct psychopathic traits.

## Introduction

Among children with conduct problems, a subgroup of children who present with a constellation of personality features, referred to as psychopathic traits, show greater risk for aggression and antisocial behavior and other long-term negative outcomes. Most modern conceptualizations of psychopathy specify between two and four dimensions of distinct psychopathic traits, including a narcissism dimension, a callousness dimension, and an impulsivity dimension (Frick & Hare, 2001; Salekin, 2016, 2017). Conceptualizations of psychopathy as early as Cleckley (1976) and Karpman (1941) have described a characteristic absence of fear among those with elevated psychopathic traits, while more recent theoretical accounts have emphasized a so-called ‘low fear’ hypothesis of psychopathy (Lykken, 1957). This characteristic lack of fear may be associated with a lack of concern for the consequences of one’s actions, including the potential for punishment or injury, and may therefore account for the relatively high incidence of aggression and antisocial behavior in these children (Cardinale et al., 2020). However, despite some support in favor of the ‘low fear’ hypothesis of psychopathy, variability in defining and measuring fear, and inconsistent findings on the relationship with distinct psychopathic traits, means that this relationship in children remains somewhat unresolved (Hoppenbrouwers et al., 2016; Sylvers et al., 2011).

Heterogeneity within the psychopathy construct is highlighted by differential associations of the narcissism, callousness, and impulsivity dimensions with an absence of the conscious experience of fear, or reduced autonomic reactivity to threatening or fear-inducing stimuli (Hoppenbrouwers et al., 2016). The method of assessment of fearfulness or fear-related responding in psychopathy research has varied widely, with the majority of studies focusing on self-reported feelings of fear, the ability to recognize and understand fear in others (e.g., fearful facial expression recognition), or the capacity to form learned aversive associations between a neutral and threatening stimulus (Hoppenbrouwers et al., 2016). However, others have relied on the use techniques for assessing reactivity of the autonomic nervous system (ANS) to a threatening or fear inducing stimulus as an index of ‘fearfulness’. Physiological changes that accompany responses to affective stimuli are mediated by the relative actions of the two branches of the ANS: the sympathetic nervous system (SNS), and the parasympathetic nervous system (PNS). Increases in SNS activity are associated with increases in heart rate and greater expenditure of energy, whereas increases in PNS activity are associated with reductions in heart rate and increased conservation of resources. The skin conductance response (SCR) represents an important and reliable indicator of SNS activity. When exposed to stress, a surge of sympathetic activation serves to increase cardiovascular output and skin conductance (Berntson et al., 1991). Importantly, SCRs may vary depending on the nature of the task (Stern et al., 2000), with an increase in SCR indicative of an increase in attention, an increase in emotional arousal (e.g., through an increase in fear, threat, or excitement), or an increase in effortful-control (Critchley, 2002). Although some studies have examined associations between distinct psychopathic traits and SCRs to threatening stimuli in adults, very few studies have examined these relationships in children.

Some of the earliest studies of the relationship between psychopathy and SCR to threat in children focused on differences between children categorized as high versus low psychopathy. For example, children who scored highly for psychopathic traits have been found to show reduced electrodermal responses to threatening stimuli compared to children with low psychopathic traits, while correlational analyses suggested that these reductions were driven by higher factor one (narcissistic/callous-unemotional) and total psychopathy scores, but not factor two (impulsivity) scores (Blair, 1999). Similar results were also reported in another extreme groups design, this time comparing anticipatory SCRs, where it was again found that children in the high psychopathic traits group showed electrodermal hyporeactivity compared to a control group of children with low psychopathic traits (Fung et al., 2005). However, because of differences in antisocial behavior between groups, it remained unclear whether or not these differences were attributable to elevated psychopathic traits, or were more simply a reflection of differences in antisociality more generally.

Several more recent studies have built on these early findings by taking a dimensional approach to understanding the relationship of psychopathic traits with SCR. The majority of these studies have reported reduced SCR in children with higher narcissistic features (variably termed interpersonal or manipulative/deceitful), but not callousness or impulsivity features, during a passive auditory task (Isen et al., 2010), during anticipation of signaled white noise bursts (Wang et al., 2012), or during anticipation and in response to aversive stimuli (Wang et al., 2015). Moreover, across all three studies, these results were only found in boys, but not girls, suggesting that electrodermal hyporeactivity may represent a biological marker of a manipulative and deceitful orientation in males (Isen et al., 2010; Wang et al., 2012, 2015).

However, not all studies have revealed consistent findings. For example, in a sample of 101 young adults, Fanti and colleagues showed that narcissism, but not callousness or impulsivity, was associated with reductions in baseline SCR (Fanti et al., 2017). Yet, all of the trait dimensions were unrelated to SCR reactivity (Fanti et al., 2017), suggesting narcissism is related to a dampened resting arousal that does not generalize to dampened reactivity to threat. Contrasting results have also been reported in a comprehensive study of physiological reactivity in 56 juvenile male offenders, ages 13 to 18 years old, recruited from a juvenile court and detention facility (MacDougall et al., 2019). In this study, narcissism was associated with heightened unsignaled anticipatory SCR, while callousness was associated with smaller unsignaled anticipatory SCR (MacDougall et al., 2019). Neither of these trait dimensions were associated with signaled anticipatory responses, nor signaled or unsignaled reactivity (MacDougall et al., 2019); further, there were no significant relations of daring-impulsive or antisocial psychopathic traits with SCR. Thus, taken together with earlier findings, the pattern of associations between psychopathy dimensions with autonomic reactivity remains relatively unclear.

The studies summarized above varied in terms of assessment of psychopathic traits, with most studies employing measurement tools with a two-factor solution. Although these results are revealing about the relationship of psychopathic with the functioning of the ANS, it is now widely accepted that child psychopathy is composed of three trait dimensions, namely narcissism, callousness, and impulsivity (Frick & Hare, 2001). Further, these studies varied in terms of the measurement of SCR, reporting on anticipatory SCR and event related SCR to a variety of aversive stimuli.

A further dimension that could affect SCRs to threatening stimuli is trait anxiety. Although the relationship of psychopathy with trait anxiety has been shown to vary across different psychopathy trait dimensions (Blonigen et al., 2010), it is notable that psychopathic traits in adolescent males and females co-occur with anxiety at a higher rate than expected (Kubak & Salekin, 2009). Of importance, fear and anxiety are distinguishable at a conceptual and neurobiological level (Sylvers et al., 2011), and it is theoretically plausible that they could show opposing relations with a particular psychopathy trait dimension (Gillespie et al., 2015). Thus, it is important to consider not only the differential relationships of fear and anxiety with psychopathy dimensions, but also whether or not fear and anxiety interact in their relation to psychopathic traits.

### Current Study

In this study, we examined whether fear and anxiety act in concert or reciprocally to explain variance in narcissism, callousness, and impulsive psychopathic traits in adolescence. We examined SCR to the discrete event of a rollercoaster drop using virtual reality (VR) in a sample of youths enrolled in a non-mainstream school designated for children with challenging emotional and behavioral difficulties. The rollercoaster drop was used to provide a sudden and fear-inducing event while SCR was used to measure changes in autonomic arousal that were in close temporal proximity to the event. Event-related SCR (which indexes phasic activation) to a discrete threat has been found to be more useful in designating youths with conduct problems than tonic levels of ANS functioning (Fanti et al., 2019). We used the teacher-reported Antisocial Process Screening Device (Frick & Hare, 2001) to examine the psychopathy-linked factors of narcissism, callousness, and impulsivity, and we examined their association with SCR (fear response) and self-reported anxiety. Based on theory and research, we hypothesized that a ‘double-dose’ of low SCR and low anxiety to the fear-inducing event would be associated with the highest levels of psychopathy. We expected this relationship to be present for both psychopathy-linked narcissism and callousness, but not impulsivity. Indeed, we predicted that higher impulsivity may even be associated with greater SCR to the fear-inducing event and heightened anxiety, consistent with earlier work showing that callousness and impulsivity often show opposing relations with ANS reactivity to threatening stimuli (Benning et al., 2005; Fanti et al., 2016, 2017; Fowles & Dindo, 2006), and that disinhibition is associated with heightened anxiety (Derefinko, 2015).

## Method

### Participants

Seventy-five adolescents were recruited from social, emotional and behavioral difficulty (EBD) schools. These are non-mainstream schools where there are smaller classes to allow teachers and behavior management specialists to deal with emotional and behavior challenges. Participants were predominantly male (*n* = 61), White British (96%), and aged between 11 and 16 (*M* = 14.0, *SD* = 1.4). Based on school records, 23% had lived in care, 34% had a history of abuse, 52% had a diagnosis of Attention Deficit Hyperactivity Disorder (ADHD), 5% had an Autism Spectrum Disorder (ASD), 5% had a diagnosis of depression and a history of self-harm, and 2% had Reactive Attachment Disorder. Participants’ legal caregivers gave consent for the young person to take part, and the participant assented to be involved in the study.

### Procedure

Ethical approval was given by the psychology subcommittee of the Faculty of Science at the University of Durham. Four schools in the North East of England were included in the recruitment process. Each school varied on recruitment success rate (71%, 87%, 60%, 42%). Information sheets and consent forms were sent home to caregivers, and only those children who had returned a signed consent form were able to participate in the study. The experiment took place in a quiet room within each school. Electrodes were placed on participants skin at the start of the testing session, following the provision of consent. Self-report questionnaires were completed by the participant prior to the experiment to accommodate a stabilization period for the electrodes. Participants completed a 3-min rest period where they were asked to sit still and try to relax. The VR headset was then placed on the participant’s head, at which point the child was asked to describe the VR surroundings. This allowed the participant to become familiar with the surroundings (e.g., sitting on a roller coaster) and provided confirmation that the participant could see the display. The roller coaster lasted for 90 s. Next, participants were introduced to a control resting condition, which was a sunny garden set in Tuscany, Italy. Participants were asked to sit still and relax for 3 min. Participants wore headphones during the roller coaster and control condition. All participants received a chocolate bar for completing the study. The form tutor (home-room teacher) for each participant was asked to complete the measure of psychopathic traits. Form tutors typically have the most contact with their tutees and would receive behavior reports from other staff. We considered that these tutors were therefore best placed to provide a reliable assessment.

#### **Skin Conductance Response**

**Fig. 1:**
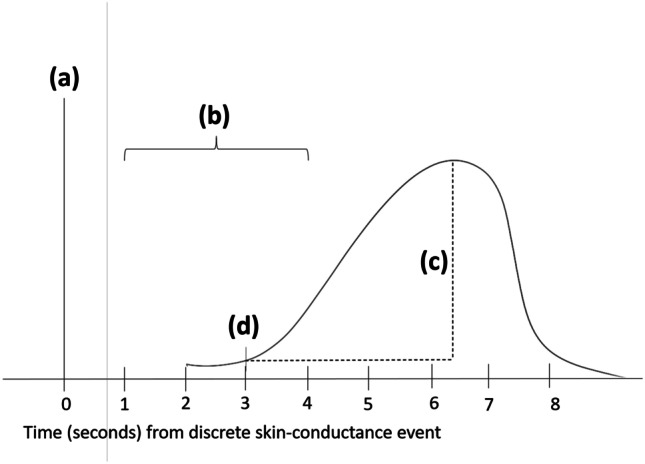
Timeline for event-related analysis of skin conductance. Note **a** is onset of event (top of rollercoaster when drop height is revealed; **b** marks the 1–4 s time window when any increase (over .05 microsiemens) in skin conductance was taken as the onset **d** of an event-related SCR; **c** was taken as the amplitude of the event-related SCR

In order to measure skin conductance, two Ag–AgCl electrodermal conductance electrodes were attached to the two middle phalanges of the non-dominant hand. Isotonic gel was applied prior to the Velcro adhesive collars being secured. Data were recorded using Biopac MP150 with BioNomadix module transmitter (MP150-BIOPAC Systems Inc., Goleta, CA), and sampled at 500 Hz. Data were reduced to 250 Hz and analyzed offline, using Biopac’s Acknowledge 4.3 software. Skin conductance was recorded using a low-pass filter of 1 Hz and a gain of 5 µS/V. The waveform was smoothed at 1000 samples. SCR was calculated by examining the peak change in skin conductance (0.05 µs or greater) within a latency window of 1 to 4 s, measured from reaching the height of the drop in the roller coaster. Only one value was below 0.05 µs and this was scored as zero. All other values ranged from 0.05 (one participant) to 1.88, suggesting that almost all participants responded to the roller coaster drop. Figure 1 illustrates the timing of responses that were coded. We measured event-related SCR, since it allows for measuring discrete responses following a fearful event.

#### **Fear-Induction**

To safely measure emotional and physiological reactivity to a fear inducing event, participants experienced a 90 s VR roller coaster. A roller coaster was selected as it is age and ethically appropriate to administer to children and adolescents for inducing a fearful response. Furthermore, in support of a roller coaster being a marker for fear (or fearlessness), the most widely used self-report measures often include roller coaster items (see the Fear Survey Schedule and the Situated Fear Questionnaire; Arrindell et al., 1984). The VR headset, the Oculus Rift, has an 18 cm 3D screen (allowing for 100 degrees of direct view) with low latency 360-degree head tracking capabilities. The headset is comfortable and lightweight, which makes the headset suitable for ages 7 years and up. Participants wore noise cancelling headphones while they were wearing the headset. The roller coaster video (RiftCoaster; Oculus, 2013) lasted for 90 s, with steep drops, tunnels, turns, and jumps. We examined SCR to the first drop.

#### **Psychopathic Traits**

The Antisocial Process Screening Device (APSD; Frick & Hare, 2001) was completed by the child’s teacher/form-tutor to measure psychopathic traits. The APSD is a self-report derivative of the Psychopathy Checklist-Revised (Hare, 2003), used to measure psychopathic traits in children and adolescents, from both forensic and community populations. The APSD consists of 20-items, with each item rated on a Likert scale from 0 *(Not at all true*) to 2 *(Definitely true)*. The APSD includes three subscales, with seven items measuring narcissism (e.g., “uses or “cons” other people to get what s/he wants”), six items measuring callousness (e.g., “does not show feelings or emotions”), and five items measuring impulsivity (e.g., “Engages in risky or dangerous activities”), and. The callousness scale includes five items that were reverse coded (e.g., “Is concerned about the feelings of others”). In the present sample, the Cronbach’s alpha coefficient for the APSD total score (0.88), and the three dimensions (narcissism = 0.84; callousness = 0.62; impulsivity = 0.75) suggests adequate reliability that is consistent with prior research. The callousness subscale showed the lowest reliability out of the three subscales, which has previously been shown across self- and parent-reports (Essau et al., 2006; Muñoz & Frick, 2007).

#### **Anxiety**

The Behavior Assessment Scale for Children, 2nd edition (BASC-2; Reynolds & Kamphaus, 2004) was used to measure anxiety. The BASC-2 is a standardized and norm-referenced self-report rating scale that is widely used among clinicians and researchers. The BASC-2 measures emotional and behavioral functioning and self-perceptions held by children and adolescents. There is robust support for the reliability and validity of the BASC-2 in adolescent samples (Frick et al., 2010). The self-report scale takes 20 min to complete, and captures levels of, anxiety, attention problems, attitudes to school/teachers, atypicality, depression, hyperactivity, interpersonal relations, locus of control, relations with parents, self-esteem, self-reliance, sense of inadequacy, and social stress (Reynolds & Kamphaus, 2004). Standardized *t*-scores only for anxiety were used in the present study.

#### **Experience of VR**

We asked participants whether they had ever been on a roller coaster. Thirteen of the 75 adolescents who took part had never been on a roller coaster. Participants were also asked to respond to the following questions using a five-point Likert scale: “Did you enjoy the roller coaster? How much did your experience in the virtual world seem like your real-world experience? How much were you totally involved/absorbed in what was going on?”. The average response values were 2.9, 2.3, and 2.6 respectively. The median was 3 (quite a lot) for enjoyment, 2 (a bit) for matching reality, and 3 (quite a lot) for absorption.

### Data Analysis Plan

Descriptive statistics and correlations were conducted using jamovi 1.1.9.0 (The jamovi project, 2019) to examine the zero-order bivariate associations among the main study variables. Regression analyses were conducted in Mplus 7.3 (Muthén & Muthén, 2012) using manifest variables with fully saturated models. We used nested models to account for the hierarchical nature of the regressions. Because full-information maximum-likelihood models with robust standard errors were used, Satorra-Bentler scaled chi-square difference test for MLR (Asparouhov & Muthén, 2010) were used to understand differences between the steps of the regression. Regression analyses were also supported by the additional R modules *car* (Fox & Weisberg, 2018) and *emmeans* (Lenth, 2018) in jamovi 1.1.9.0 (The jamovi project, 2019), which has a core language in R (RCore Team, 2018). These were used to conduct post-hoc tests of the moderation of SCR. Two participants refused to complete the roller coaster, so they were not included in the regression analyses. Additionally, only 59 participants completed the self-report anxiety measure. Thus, regression analyses that included anxiety were based on this sub-sample of participants.

To test whether there was an interaction between anxiety and SCR to the roller coaster drop in predicting psychopathic traits, we ran a hierarchical regression with narcissism, callousness, and impulsivity trait scores as dependent variables in the model. The presence of a significant interaction would suggest that the anxiety-psychopathy association was moderated by SCR. Because 32% of the sample were taking medication, and this can influence cardiovascular psychophysiological measures (e.g., Ritalin), current medication use was controlled for in the analyses. Step 1 of the regression model regressed the three psychopathy subscales on medication, anxiety, and SCR. The covariation among the psychopathy subscales was modeled as part of the regression to control for the covariance among narcissism, callousness, and impulsivity. Step 2 included the multiplicative term of anxiety * SCR to the drop as a statistical predictor. Following a significant Satorra-Bentler chi-square difference test, we conducted post-hoc testing of the significant moderation effect in jamovi. This was done by solving the regression equation by inserting values of SCR that were one standard deviation below the mean, at the mean, and one standard deviation above the mean (see Holmbeck, 2002). The simple slopes were calculated and plotted using the *medmod* package for R within jamovi. Bootstrapped standard errors were calculated with 10,000 samples.

## Results

Table 1 shows the descriptive statistics. SCR to the drop was positively skewed (*Z* = 5.13). After performing a square root transformation, skewness was reduced to 0.595 (*SE* = 0.281). Examination of violin plots and boxplots revealed that the variable approximated a normal distribution with no outliers. Means and standard deviations are shown in Table 1 and SCR mean and standard deviation are given for the non-transformed variable to aid comparison with other studies.

**Table 1:** Descriptive statistics and zero-order correlations for main study variables

	**1**	**2**	**3**	**4**	**5**	**6**	**7**
1. SCR to drop	—						
2. APSD total	-0.02	—					
3. APSD NAR	0.12	0.89***	—				
4. APSD CU	-0.13	0.64***	0.37**	—			
5. APSD IMP	-0.01	0.82***	0.65***	0.32**	—		
6. Anxiety	0.13	0.18	0.27*	-0.06	0.19	—	
7. Gender (1 = female)	-0.02	-0.04	0.03	-0.25*	0.00	0.22	—
Mean	0.46	20.90	6.78	5.84	6.58	53.50	Male = 84%
SD	0.41	7.82	3.76	2.27	2.39	11.60	

*APSD* Antisocial Process Screening Device (Frick & Hare, 2001), *NAR* narcissism, *CU* callous-unemotional, *IMP* impulsivity

*p < .05, **p < .01, ***p < .001. Associations with gender were conducted with Spearman’s rank correlations

Correlations noted in Table 1 showed that, although not significant, greater SCR to the drop was associated with higher levels of narcissism and lower levels of callousness. Narcissism was positively and significantly associated with child-reported anxiety, but the positive association between impulsivity and anxiety did not reach significance; nor did the negative association between callousness and anxiety. There was a small positive association between SCR and anxiety, but it was not significant. Of importance, there were no significant associations between SCR and reports of enjoyment of the roller coaster experience (*r* = 0.18, *p* = 0.156), VR matching reality (*r* = 0.19, *p* = 0.140), or absorption in the VR experience (*r* = 0.03, *p* = 0.817). This suggests that the SCR to the drop was not influenced by positive affect or any variation in participants’ perceptions of the roller coaster as matching reality. Teacher reports indicated that boys showed higher levels of callousness, but because of the low number of girls recruited to this study we did not control for gender.

### Are There Significant Associations of Psychopathy Swith SCR and Anxiety and Their Interaction?

**Fig. 2:**
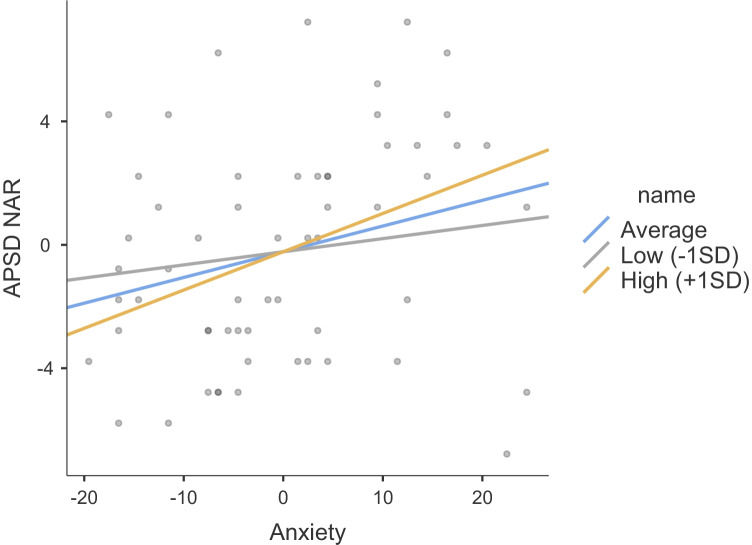
Plot showing the moderating effect of fear (SCR to drop) on the association between anxiety and narcissism (APSD NAR)

**Fig. 3:**
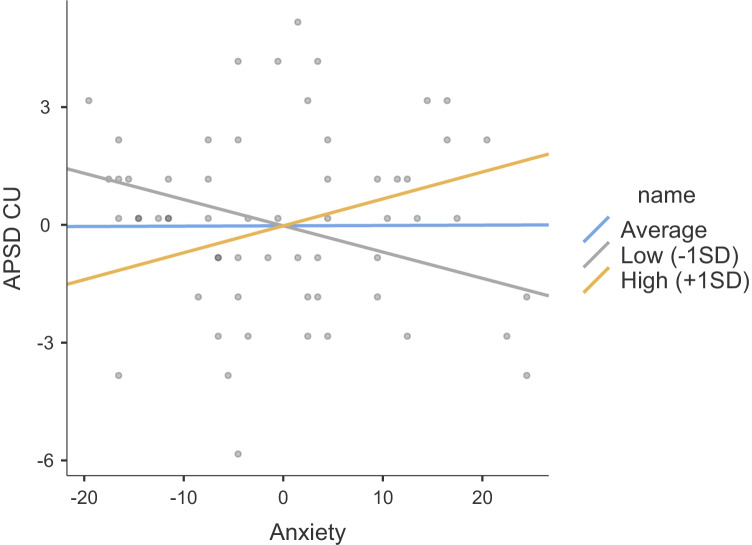
Plot showing the moderating effect of fear (SCR to drop) on the association between anxiety and CU traits (APSD CU)

**Fig. 4:**
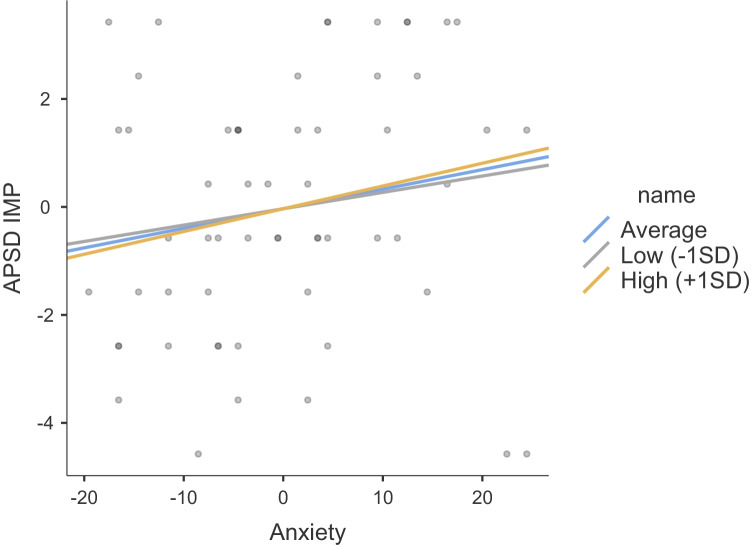
Plot showing the moderating effect of fear (SCR to drop) on the association between anxiety and impulsivity (APSD IMP)

The results of regression models including SCR to the drop, anxiety and their interaction as predictors of psychopathic traits are summarized in Table 2. These included unstandardized beta estimates, standard errors, and 95% confidence intervals. None of the regression estimates were significant for Step 1, which included medication, anxiety and SCR. When comparing the two models (the nested model without the interaction and the model with the interaction), there was a significant change in the model fit (Satorra-Bentler chi-square difference test for -2LL = 9.62, df = 3, *p* < 0.025). Figures 2, 3, and 4 show the interaction plots for all psychopathy subscales of narcissism, callousness, and impulsivity, respectively. Only the beta value for the interaction predicting callousness was significant, and there was a 10% increase in R-square from Step 1 to 2. Indeed, in examining the interaction plot of narcissism, all plot lines show a positive slope, suggesting that SCR does not moderate the association between anxiety and narcissism. Post-hoc testing of the simple slopes predicting callousness showed that the simple slope of anxiety statistically predicting levels of callousness was significant at low levels (-1SD) of SCR (estimate = -0.07, SE = 0.03, 95%CI = -0.12, -0.004, *Z* = -2.27, *p* = 0.023), but not at the mean of SCR (estimate = 0.001, SE = 0.03, 95%CI = -0.05, 0.05, *Z* = 0.03, *p* = 0.973), or at high levels (+ 1SD) of SCR (estimate = 0.07, SE = 0.04, 95%CI = -0.01, 0.15, *Z* = 1.72, *p* = 0.085). A double-dose of low SCR to the drop (indicative of low fear) and low anxiety was associated with the highest levels of callousness.

**Table 2:** Hierarchical regressions regressing psychopathy subscales on levels of anxiety and fear (SCR to the roller coaster drop) and their interaction, while controlling for medication use

	APSD NAR			APSD CU			APSD IMP		
**Step 1**	**beta**	**95%CI**	**SE**	**beta**	**95%CI**	**SE**	**beta**	**95%CI**	**SE**
Anxiety	0.08	-0.01, 0.17	0.05	-0.01	-0.06, 0.04	0.03	0.04	-0.03, 0.10	0.03
Meds:									
Yes – No	-0.86	-2.67, 0.95	0.92	-0.65	-1.80, 0.51	0.59	-0.18	-0.43, 1.07	0.64
SCR Drop	1.66	-2.31, 5.62	2.02	-1.29	-3.79, 1.21	1.27	0.61	-1.44, 2.66	1.05
**Step 2**									
Anxiety	-.001	-0.20, 0.20	0.10	-0.15*	-0.25, - 0.05	0.05	0.03	-0.11, 0.16	0.07
Meds:									
Yes – No	-0.72	-2.57, 1.12	0.95	-0.41	-1.59, 0.77	0.60	-0.16	-1.43, 1.10	0.65
SCR Drop	-5.94	-22.39, 10.50	8.39	-15.00*	-24.43, - 5.56	4.81	-0.41	-11.38, 10.55	5.60
SCR X Anxiety	0.14	-0.16, 0.43	0.15	0.25*	0.09, 0.41	0.08	0.02	-0.17, 0.21	0.10
Step 1 *R*^2^		.10			.04			.04	
Step 2 *R*^2^		.12			.14			.04	

*APSD* Antisocial Process Screening Device, *NAR* narcissism, *CU* callous-unemotional traits, *IMP* impulsivity, *SCR* skin conductance response

^*^p < .05

## Discussion

Psychopathy is a multidimensional construct consisting of narcissistic, callous, and impulsive tendencies (Salekin, 2016, 2017). These trait dimensions have been found to show differential relations with indices of emotion processing and physiological reactivity, with a particular emphasis on the experience of fear. In the present study, we aimed to examine whether fear and anxiety act in concert or reciprocally to explain variance in narcissism, callousness, and impulsive psychopathic traits in adolescence. We used a measure of physiological reactivity to a VR roller coaster drop event as an index of fear reactivity, and asked participants to self-report their experiences of anxiety. In line with expectations, we found that callousness was related to a double-dose of low SNS reactivity, in the form of low SCR to a fear-inducing drop on a VR roller coaster, and low anxiety. Although zero-order associations showed that narcissism was positively associated with anxiety, there were no significant relations of either narcissism or impulsivity with SCR. The interaction effects between anxiety and SCR to the drop in predicting narcissism and impulsivity were also non-significant. Although narcissism and impulsivity showed nonsignificant positive associations with anxiety, these associations were not moderated by SCR. These findings further our understanding of psychopathy dimensions and affective responses to threat, both in terms of peripheral psychophysiological responding and in terms of trait-anxiety.

Prior to interpreting the findings fully, it is necessary to outline the mechanisms of SCR. SCR has diverse mechanisms as an index of SNS reactivity. The VR task in the present study involved sitting on a VR roller coaster, watching the roller coaster in first-person perspective while it ascended slowly to the top of a mountain, and then watching it descend quickly through a fantasy valley. An increase in SC (i.e., SCR) could indicate an increase in attention, an increase in emotional arousal (through an increase in fear, threat, or excitement), or an increase in effortful control that might lead to an increase in peripheral SNS activation. Since we marked the timing of the event across all participants, we ensured that physiological responses (i.e., the SCR) were locked to the drop event. However, the emotional salience of the event can also vary based on novelty, familiarity, personal significance, potential anticipation of reward, memory recall, and attentional and cognitive effort (Critchley, 2002). The results that we report here suggest that the associations we found are unlikely to reflect enjoyment or absorption with the VR roller coaster, as there were no significant associations between SCR to the drop and enjoyment and absorption with the stimulus.

SCR was chosen for its role in measuring fearful arousal. Prior research has shown that low event-related SCR was associated with greater ventromedial prefrontal cortex (vmPFC) activation (Zhang et al., 2014), suggesting that vmPFC activation helps to curb fear-related responses (Nili et al., 2010). Additionally, while SCR appears to be negatively associated with activity largely in the posterior vmPFC, positive associations with SCR were mainly found in areas of the brain associated with anxiety (Zhang et al., 2014). Based on the above, we expect that SCR to the roller coaster drop reflects a measure of threat or fearful arousal.

Our results for the narcissism dimension are consistent with earlier work showing that participants who score higher on the narcissism dimension experience greater anxiety (Barry & Malkin, 2010; Lee-Rowland et al., 2020). Lee-Rowland et al. (2020) proposed that youths with high levels of narcissism may exert superiority over others and protect their self-image due to a sense of insecurity and high levels of anxiety. This tendency to exert superiority over others may lead to increased aggressiveness and attempts to dominate others (Barry et al., 2007; Golmaryami & Barry, 2010; Washburn et al., 2004). The absence of an association between narcissism and SCR is surprising in light of previous research showing a reduction in SCR in anticipation of, and in response to, an aversive stimulus (Isen et al., 2010; Wang et al., 2012, 2015). Recently, for example, MacDougall et al. (2019) showed that grandiose/manipulative traits (roughly equivalent to psychopathy-linked narcissism) were associated with heightened SCR during anticipation of, and in response to, an aversive event (white noise). However, previous studies have predominantly tested these effects in samples of boys, and our findings suggest that these effects may differ in mixed samples of boys and girls. It is also noteworthy that the task used in the present study could be interpreted as thrilling rather than aversive, which may elicit a different pattern of relations with physiological responding.

In contrast to the results for narcissism, callousness was related to measures of emotional experience that indicate low peripheral reactivity in response to a fear inducing stimulus as well as low anxiety. Our results are largely consistent with the findings of MacDougall et al. (2019). In their study, callousness was related to lower SCR during the anticipatory period where youths were not alerted to the subsequent arrival of the aversive stimulus (MacDougall et al., 2019). Yet, our own results go beyond these findings to also show that anxiety was associated with callousness, but only at low levels of SCR to the fear inducing event. Thus, it is possible that youths who are more callous might down-regulate their arousal in the face of fear or threat and could be less prone to feelings of anxiety. Indeed, Thomson et al. (2019) found evidence for heightened emotion regulation for youths high on callous-unemotional traits when measuring PNS activity (i.e., augmentation of respiratory sinus arrhythmia) while experiencing the entire duration of a real-3D TV and VR roller coaster. This has led to proposals regarding the usefulness of measuring ANS activity or reactivity to aid the assessment of callousness and interventions targeting socioemotional functioning during social affiliation (Wagner & Waller, 2020). Our findings reported here may help to explain a link between callousness and insensitivity to punishment (Muñoz & Frick, 2012), whereby youths who fail to respond to fear or threat with a sympathetic response may find it hard to remember and learn from negative events, leading to difficulties in early socialization (Fowles & Kochanska, 2000; Kochanska, 1993).

Our results showed that the impulsivity dimension was unrelated to either SCR to the fear inducing event or anxiety. These findings are largely consistent with earlier work, with most reports documenting the absence of statistically significant association between SCR and the impulsivity features of psychopathy in children (Isen et al., 2010; MacDougall et al., 2019). Taken together, these findings suggest that electrodermal reactivity may not represent a reliable, biological marker of impulsivity. The absence of an association between impulsivity and SCR in study might also reflect the nature of the stimulus used in this study, which employed a thrilling roller coaster drop, and earlier work that has presented anger inducing stimuli and has required a behavioral response (Tangney et al., 2004).

There are several limitations to note in interpreting our findings. First, although this was a sample with emotional and behavioral difficulties, we did not perform any diagnostic testing for conduct problems. It is important to note that research suggests that findings differ for those with callous-unemotional traits in the presence versus absence of conduct disorder (Fanti et al., 2017, 2018), and diagnoses of conduct disorder should therefore be considered in future research. Our sample also showed high rates of ADHD, and some participants had existing diagnoses of ASD, reported taking medication, and had histories of abuse. Although rates of comorbid disorders and histories of medication and abuse are common in samples of children with emotional and behavioral difficulties, and our sample is therefore representative of the realities of working with these populations, these factors may make replication in other samples more difficult. Other problems in affective functioning, for example low or depressed mood, were not assessed here but should be examined in future studies.

Other limitations related to the measures that we used. For example, our measure of callousness was only moderately internally consistent. Despite some doubts in the present sample, this subscale has shown stability over time, as would be expected for a measure of personality traits (Muñoz & Frick, 2007), and shows prospective relations with antisocial behavior over and above the other psychopathy trait dimensions (Muñoz & Frick, 2007). A further limitation arises from the use of a single drop event in the VR based roller coaster, meaning that our analyses were based on a single-item measure of SCR. Although data from other drop events during the roller coaster could be used, problems with habituation led to little variance in reactivity to the drop. This task was also used to capture reactivity to a fear inducing event, and the presence versus absence of fear has been linked in particular with the callousness features of psychopathy (Cardinale et al., 2020). Thus, other VR settings that elicit emotions other than fear or the thrill and excitement of a roller coaster may be associated with different patterns of physiological reactivity, and different relations with psychopathic traits. Finally, we had a relatively small sample owing to the difficulty in conducting psychophysiological studies using VR within schools. However, one of the strengths of the study is the relationships with school headteachers and teachers. The cooperation with multiple school officials also meant that recruitment of a specialist group of youths was successful.

The VR context provided us with an opportunity to examine children’s psychophysiological responses under more realistic conditions compared to using film clips, static pictures, or contrived threatening events (where there are no real consequences). It would have been difficult to collect psychophysiological responses on a real-life roller coaster, but a fantasy world can easily be created that leads to physiological sensations in VR. One noteworthy prior study has also examined responses to a real-life fear-inducing event. During five emotionally evocative life events (e.g., getting inoculated), adolescent youths with psychopathic traits showed specific reductions in self-reported fearfulness (Marsh et al., 2011). However, in that study, the authors did not examine the consistency of self-reported fear with physiological reactivity. The benefits of using VR have been argued to be “making the impossible, possible” (to create environments that would be hard or impossible to recreate outside of the realm of imagination), “making the possible, safer” (one can explore dangerous or threatening scenarios in complete safety) and “making the possible more safe, more practical and more effective” (Negen, 2018). Thus, using VR could have intervention and research implications, especially when used in combination with psychophysiological techniques to understand arousal and affect regulation processes under more life-like conditions, as has been attempted for borderline personality traits (McLachlan et al., 2021).

Youths who score highly on different psychopathy trait dimensions may present cause for concern in different ways. By taking a dimensional approach to understanding the relationship of narcissistic, callous, and impulsive psychopathic traits with SCR, we were able to demonstrate differential relations of psychopathic traits with fear reactivity and self-reported anxiety. In sum, we found that narcissism, in this multi-health problem group, was associated with anxiety but not with the response to a thrilling event of a roller coaster, while callousness was related to a dampening of emotions across measures of fear reactivity and anxiety, and impulsivity was not significantly associated with either SCR or anxiety. The finding that higher levels of the affective component of psychopathy (i.e., callousness) were related to low anxiety and low SCR may be of importance for understanding the aggressive and antisocial behaviors associated with callousness, but the uniqueness of our sample means that more research is needed. As noted by Fanti (2018), the psychopathy trait dimensions are differentially associated with emotional and physiological reactivity, and our findings suggest that simultaneous measurement of internal emotional states may also be of considerable importance. Future work should also consider that ANS activation been found to be inconsistently related to brain activation across peripheral organs (Eisenbarth et al., 2016), and so triangulating across different peripheral ANS measures (i.e., SCR, respiratory sinus arrhythmia, etc.) may be fruitful. Using VR may be one way to not only test emotional responses to frightening events, but it may also be of potential use in interventions.

## References

[CR1] Arrindell WA, Emmelkamp PM, van der Ende J (1984). Phobic dimensions: I Reliability and generalizability across samples, gender and nations. Advances in Behaviour Research and Therapy.

[CR2] Asparouhov, T., & Muthén, B. (2010). Computing the strictly positive Satorra-Bentler chi-square test in Mplus. *Mplus Web Notes*, *12*.

[CR3] Barry CT, Malkin ML (2010). The relation between adolescent narcissism and internalizing problems depends on the conceptualization of narcissism. Journal of Research in Personality.

[CR4] Barry TD, Thompson A, Barry CT, Lochman JE, Adler K, Hill K (2007). The importance of narcissism in predicting proactive and reactive aggression in moderately to highly aggressive children. Aggressive Behavior.

[CR5] Benning SD, Patrick CJ, Iacono WG (2005). Psychopathy, startle blink modulation, and electrodermal reactivity in twin men. Psychophysiology.

[CR6] Berntson GG, Cacioppo JT, Quigley KS (1991). Autonomic determinism: The modes of autonomic control, the doctrine of autonomic space, and the laws of autonomic constraint. Psychological Review.

[CR7] Blair RJR (1999). Responsiveness to distress cues in the child with psychopathic tendencies. Personality and Individual Differences.

[CR8] Blonigen DM, Patrick CJ, Douglas KS, Poythress NG, Skeem JL, Lilienfeld SO, Krueger RF (2010). Multimethod assessment of psychopathy in relation to factors of internalizing and externalizing from the Personality Assessment Inventory: The impact of method variance and suppressor effects. Psychological Assessment.

[CR9] Cardinale EM, Ryan RM, Marsh AA (2020). Maladaptive fearlessness: An examination of the association between subjective fear experience and antisocial behaviors linked with callous unemotional traits. Journal of Personality Disorders.

[CR10] Cleckley, H. (1976). The mask of sanity . St. Louis, MO. *V. Mosby*.

[CR11] Critchley HD (2002). Electrodermal responses: What happens in the brain. The Neuroscientist.

[CR12] Derefinko, K. J. (2015). Psychopathy and Low Anxiety: Meta-Analytic Evidence for the Absence of Inhibition, Not Affect. *Journal of Personality, 83*(6), 693–709. 10.1111/jopy.12124 10.1111/jopy.12124 25130868

[CR13] Eisenbarth H, Chang LJ, Wager TD (2016). Multivariate brain prediction of heart rate and skin conductance responses to social threat. The Journal of Neuroscience.

[CR14] Essau CA, Sasagawa S, Frick PJ (2006). Callous-unemotional traits in a community sample of adolescents. Assessment.

[CR15] Fanti KA (2018). Understanding heterogeneity in conduct disorder: A review of psychophysiological studies. Neuroscience and Biobehavioral Reviews.

[CR16] Fanti KA, Eisenbarth H, Goble P, Demetriou C, Kyranides MN, Goodwin D, Cortese S (2019). Psychophysiological activity and reactivity in children and adolescents with conduct problems: A systematic review and meta-analysis. Neuroscience and Biobehavioral Reviews.

[CR17] Fanti KA, Kyranides MN, Georgiou G, Petridou M, Colins OF, Tuvblad C, Andershed H (2017). Callous-unemotional, impulsive-irresponsible, and grandiose-manipulative traits: Distinct associations with heart rate, skin conductance, and startle responses to violent and erotic scenes. Psychophysiology.

[CR18] Fanti KA, Kyranides MN, Petridou M, Demetriou CA, Georgiou G (2018). Neurophysiological markers associated with heterogeneity in conduct problems, callous unemotional traits, and anxiety: Comparing children to young adults. Developmental Psychology.

[CR19] Fanti KA, Panayiotou G, Kyranides MN, Avraamides MN (2016). Startle modulation during violent films: Association with callous–unemotional traits and aggressive behavior. Motivation and Emotion.

[CR20] Fowles, D. C., & Dindo, L. (2006). A dual-deficit model of psychopathy. *Handbook of psychopathy*.

[CR21] Fowles DC, Kochanska G (2000). Temperament as a moderator of pathways to conscience in children: The contribution of electrodermal activity. Psychophysiology.

[CR22] Fox, J., & Weisberg, S. (2018). *car: Companion to applied regression*. Computer software, R package.

[CR23] Frick, P. J., Barry, C. T., & Kamphaus, R. W. (2010). Self-Report Inventories. In *Clinical assessment of child and adolescent personality and behavior* (pp. 101–139). Boston, MA: Springer US. 10.1007/978-1-4419-0641-0_6

[CR24] Frick PJ, Hare RD (2001). Antisocial process screening device: APSD.

[CR25] Fung MT, Raine A, Loeber R, Lynam DR, Steinhauer SR, Venables PH, Stouthamer-Loeber M (2005). Reduced electrodermal activity in psychopathy-prone adolescents. Journal of Abnormal Psychology.

[CR26] Gillespie SM, Mitchell IJ, Satherley R-M, Beech AR, Rotshtein P (2015). Relations of distinct personality traits with anxiety and fear: Findings from offenders and non-offenders. PLoS ONE.

[CR27] Golmaryami FN, Barry CT (2010). The associations of self-reported and peer-reported relational aggression with narcissism and self-esteem among adolescents in a residential setting. Journal of Clinical Child and Adolescent Psychology.

[CR28] Hare, R. D. (2003). Manual for the revised psychopathy checklist. In. Toronto, ON, Canada: Multi-Health Systems.

[CR29] Holmbeck, G. N. (2002). Post-hoc Probing of Significant Moderational and Mediational Effects in Studies of Pediatric Populations. *Journal of Pediatric Psychology, 27*(1), 87–96. 10.1093/jpepsy/27.1.87 10.1093/jpepsy/27.1.87 11726683

[CR30] Hoppenbrouwers SS, Bulten BH, Brazil IA (2016). Parsing fear: A reassessment of the evidence for fear deficits in psychopathy. Psychological Bulletin.

[CR31] Isen J, Raine A, Baker L, Dawson M, Bezdjian S, Lozano DI (2010). Sex-specific association between psychopathic traits and electrodermal reactivity in children. Journal of Abnormal Psychology.

[CR32] Karpman, B. (1941). On the need of separating psychopathy into two distinct clinical types: the symptomatic and the idiopathic. *Journal of Criminal Psychopathology*.

[CR33] Kochanska G (1993). Toward a synthesis of parental socialization and child temperament in early development of conscience. Child Development.

[CR34] Kubak FA, Salekin RT (2009). Psychopathy and Anxiety in Children and Adolescents: New Insights on Developmental Pathways to Offending. Journal of Psychopathology and Behavioral Assessment.

[CR35] Lee-Rowland LM, Lui JHL, Bortfeld D, Barry CT, Reiter S (2020). Internalizing problems and their impact on the relation between callous-unemotional traits, narcissism, and aggression. Aggressive Behavior.

[CR36] Lenth, R. (2018). *emmeans: Estimated Marginal Means, aka Least-Squares Means.* Computer software, R package.

[CR37] Lykken DT (1957). A study of anxiety in the sociopathic personality. Journal of Abnormal Psychology.

[CR38] MacDougall EAM, Salekin RT, Gillen CTA (2019). Adolescent psychopathy, heart rate, and skin conductance. Psychophysiology.

[CR39] Marsh AA, Finger EC, Schechter JC, Jurkowitz ITN, Reid ME, Blair RJR (2011). Adolescents with psychopathic traits report reductions in physiological responses to fear. Journal of Child Psychology and Psychiatry, and Allied Disciplines.

[CR40] McLachlan J, Mehdikhani M, Larham B, Centifanti LCM (2021). Borderline personality traits and emotion regulation strategies in adolescents: The role of implicit theories. Child Psychiatry and Human Development.

[CR41] Muñoz LC, Frick PJ (2007). The reliability, stability, and predictive utility of the self-report version of the Antisocial Process Screening Device. Scandinavian Journal of Psychology.

[CR42] Muñoz LC, Frick PJ (2012). Callous-unemotional traits and their implication for understanding and treating aggressive and violent youths. Criminal Justice and Behavior.

[CR43] Muthén, L., & Muthén, B. (2012). *Mplus user’s guide (5th ed.)*.

[CR44] Negen, J. E. (2018, April 17). *Using VR in Psychology*. Presented at the VR Workshop, University of Liverpool.

[CR45] Nili U, Goldberg H, Weizman A, Dudai Y (2010). Fear thou not: Activity of frontal and temporal circuits in moments of real-life courage. Neuron.

[CR46] Oculus, V. R. (2013). Oculus Riftcoaster (DK2). Computer software, Oculus.

[CR47] RCore Team. (2018). *R: A Language and envionment for statistical computing* . Computer software, RCore Team.

[CR48] Reynolds, C. R., & Kamphaus, R. W. (2004). BASC-2 Behavior Assessment for Children Manual.

[CR49] Salekin RT (2016). Psychopathy in childhood: Why should we care about grandiose-manipulative and daring-impulsive traits?. The British Journal of Psychiatry.

[CR50] Salekin RT (2017). Research review: What do we know about psychopathic traits in children?. Journal of Child Psychology and Psychiatry, and Allied Disciplines.

[CR51] Stern RM, Ray WJ, Quigley KS (2000). Psychophysiological Recording. Oxford University Press.

[CR52] Sylvers P, Lilienfeld SO, LaPrairie JL (2011). Differences between trait fear and trait anxiety: Implications for psychopathology. Clinical Psychology Review.

[CR53] Tangney JP, Baumeister RF, Boone AL (2004). High self-control predicts good adjustment, less pathology, better grades, and interpersonal success. Journal of Personality.

[CR54] The jamovi project. (2019). jamovi. (Version 1.1). Computer software, The jamovi project.

[CR55] Thomson ND, Gillespie SM, Centifanti LCM (2019). Callous-unemotional traits and fearlessness: A cardiovascular psychophysiological perspective in two adolescent samples using virtual reality. Development and Psychopathology.

[CR56] Wagner NJ, Waller R (2020). Leveraging parasympathetic nervous system activity to study risk for psychopathology: The special case of callous-unemotional traits. Neuroscience & Biobehavioral Reviews.

[CR57] Wang P, Baker LA, Gao Y, Raine A, Lozano DI (2012). Psychopathic traits and physiological responses to aversive stimuli in children aged 9–11 years. Journal of Abnormal Child Psychology.

[CR58] Wang P, Gao Y, Isen J, Tuvblad C, Raine A, Baker LA (2015). Genetic covariance between psychopathic traits and anticipatory skin conductance responses to threat: Evidence for a potential endophenotype. Development and Psychopathology.

[CR59] Washburn JJ, McMahon SD, King CA, Reinecke MA, Silver C (2004). Narcissistic features in young adolescents: Relations to aggression and internalizing symptoms. Journal of Youth and Adolescence.

[CR60] Zhang S, Hu S, Chao HH, Ide JS, Luo X, Farr OM, Li CR (2014). Ventromedial prefrontal cortex and the regulation of physiological arousal. Social Cognitive and Affective Neuroscience.

